# Concomitant radiosurgical and targeted oncological treatment improves the outcome of patients with brain metastases from gastrointestinal cancer

**DOI:** 10.1186/s13014-023-02383-5

**Published:** 2023-12-09

**Authors:** Farjad Khalaveh, Anna Cho, Abdallah Shaltout, Helena Untersteiner, Beate Kranawetter, Dorian Hirschmann, Philipp Göbl, Wolfgang Marik, Brigitte Gatterbauer, Karl Rössler, Christian Dorfer, Josa M. Frischer

**Affiliations:** 1 Department of Neurosurgery, Medical University of Vienna, Waehringer Guertel 18-20, Vienna, 1090 Austria; 2 Department of Neurology, Medical University of Vienna, Vienna, Austria; 3 Department of Radiology, Division of Neuro- and Musculoskeletal Radiology, Medical University of Vienna, Vienna, Austria

**Keywords:** Gamma Knife Radiosurgery, Brain metastases, Gastrointestinal cancer, Immunotherapy, Targeted therapy

## Abstract

**Background:**

So far, only limited studies exist that evaluate patients with brain metastases (BM) from GI cancer and associated primary cancers who were treated by Gamma Knife Radiosurgery (GKRS) and concomitant immunotherapy (IT) or targeted therapy (TT).

**Methods:**

Survival after GKRS was compared to the general and specific Graded Prognostic Assessment (GPA) and Score Index for Radiosurgery (SIR). Further, the influence of age, sex, Karnofsky Performance Status Scale (KPS), extracranial metastases (ECM) status at BM diagnosis, number of BM, the Recursive Partitioning Analysis (RPA) classes, GKRS1 treatment mode and concomitant treatment with IT or TT on the survival after GKRS was analyzed. Moreover, complication rates after concomitant GKRS and mainly TT treatment are reported.

**Results:**

Multivariate Cox regression analysis revealed IT or TT at or after the first Gamma Knife Radiosurgery (GKRS1) treatment as the only significant predictor for overall survival after GKRS1, even after adjusting for sex, KPS group, age group, number of BM at GKRS1, RPA class, ECM status at BM diagnosis and GKRS treatment mode. Concomitant treatment with IT or TT did not increase the rate of adverse radiation effects. There was no significant difference in local BM progression after GKRS between patients who received IT or TT and patients without IT or TT.

**Conclusion:**

Good local tumor control rates and low rates of side effects demonstrate the safety and efficacy of GKRS in patients with BM from GI cancers. The concomitant radiosurgical and targeted oncological treatment significantly improves the survival after GKRS without increasing the rate of adverse radiation effects. To provide local tumor control, radiosurgery remains of utmost importance in modern GI BM management.

## Introduction

The incidence of gastrointestinal (GI) cancer has increased over the past decades with 5.1 million newly diagnosed cases and 3.6 million deaths worldwide in 2020 [1]. It is predicted that these numbers will further increase [1]. Furthermore, despite GI cancer rarely metastasizes into the brain, the incidence of brain metastases (BM) from GI cancer has increased as well [2, 3]. This might result from the recent advent of new systemic oncological therapies and the increased availability of neuroradiological diagnostics [3]. Still, the prognosis of GI patients rapidly deteriorates once BM occur [4–7].

Standard local treatment options for BM from GI tumors comprise microsurgery and stereotactic radiosurgery (SRS) [8, 9]. Recently, SRS has become one of the most essential treatment options in patients with BM due to its efficacy and safe applicability [10, 11]. Moreover, modern oncological treatment options have significantly improved the prognosis in patients with BM from other primary tumors [12–16]. However, in contrast to other primary tumors, it appears that less attention has been paid to patients with BM from GI cancer with only few data on the prognosis of these patient cohort under immunotherapy (IT) or targeted therapy (TT) [7, 17, 18]. Therefore, we aimed to evaluate the outcome after GKRS treatment and the effect of concurrent treatment with IT or TT in patients with BM from GI cancer.

## Methods

### Study population and data evaluation

We performed a retrospective analysis of 81 patients with BM from GI and associated cancers who had been treated by at least one GKRS for at least one BM at our department between 2012 and 2020 (Table 1). As previously described, we included BM patients with GI and associated cancers, since the response to radiosurgical treatment is comparable among BM from these primary tumors [3, 19–22]. At time of BM diagnosis, the Score Index for Radiosurgery (SIR), the general and specific Graded Prognostic Assessment (GPA) and RPA classes were evaluated [6, 23–25]. The primary study endpoint was defined as death, irrespective of the cause of death. In addition, a national death register comparison was performed for data completion. Patients who were lost to follow-up were excluded from the outcome analysis but included in the descriptive analysis.

**Table 1:** Baseline characteristics of the GI-BM cohort treated with GKRS

Variable	Time of GKRS1, Total Sample (*n* = 81)	Patients with IT or TT at or after GKRS1 “IT or TT Group” (*n* = 21)*	Patients without IT or TT at or after GKRS1 “None Group” (*n* = 50)†	IT or TT vs. None Group
Age in years, median (range)	65 (35–82)	59 (41–79)	66 (35–82)	*p* = 0.011
Female/male ratio	23:58	7:14	15:35	*p* = 0.785
KPS in %, median (range)	80 (40–90)	80 (50–90)	80 (50–90)	*p* = 0.753
KPS groups				
≥80%	44 (54%)	11 (53%)	27 (54%)	*p* = 1.000
<80%	37 (46%)	10 (47%)	23 (46%)	
ECM status at time of BM diagnosis				
Yes	71 (88%)	20 (95%)	41 (82%)	*p* = 0.262
No	10 (12%)	1 (5%)	9 (18%)	
GI tumor location				
Rectum	49 (60%)	15 (71%)	28 (56%)	*p* = 0.637
Colon	11 (13%)	4 (19%)	6 (12%)	
Esophagus	9 (11%)	1 (5%)	7 (14%)	
Liver	4 (5%)	1 (5%)	2 (4%)	
Gastric	2 (3%)	0 (0%)	1 (2%)	
Pancreatic	2 (3%)	0 (0%)	2 (4%)	
Other	4 (5%)	0 (0%)	4 (8%)	
GI tumor subtype				
Adenocarcinoma	63 (78%)	17 (81%)	38 (76%)	*p* = 0.781
Squamous cell carcinoma	9 (11%)	2 (9%)	7 (14%)	
Neuroendocrine	1 (1%)	0 (0%)	1 (2%)	
Hepatocellular	4 (5%)	1 (5%)	2 (4%)	
Cholangiocellular	1 (1%)	0 (0%)	1 (2%)	
Mixed carcinoma	1 (1%)	0 (0%)	1 (2%)	
Not known	2 (3%)	1 (5%)	0 (0%)	
CNS treatment before GKRS1				
None	60 (74%)	14 (67%)	39 (78%)	*p* = 0.573
WBRT and/or fRT	2 (3%)	1 (5%)	1 (2%)	
BM resection without RT	9 (11%)	3 (14%)	4 (8%)	
BM resection with WBRT and/or fRT	10 (12%)	3 (14%)	6 (12%)	
Localization of BM at initial diagnosis				
Multiple	42 (52%)	9 (43%)	26 (52%)	*p* = 0.888
Frontal	5 (6%)	2 (9%)	2 (4%)	
Parietal	8 (10%)	3 (14%)	5 (10%)	
Central	6 (8%)	1 (5%)	4 (8%)	
Cerebellar	14 (17%)	5 (24%)	8 (16%)	
Temporal	5 (6%)	1 (5%)	4 (8%)	
Basal ganglia	1 (1%)	0 (0%)	1 (2%)	
Predicted survival after prognostic scores in months, median (range)				
GPA general	3.8 (2.6–6.9)	3.8 (2.6–3.8)	3.8 (2.6–6.9)	*p* = 0.547 *p* = 0.865 *p* = 0.536 *p* = 0.259
GPA specific	4.4 (3.1–13.5)	3.1 (3.1–13.5)	4.4 (3.1–6.9)	
RPA	4.5 (2.3–4.5)	4.5 (2.3–4.5)	4.5 (2.3–4.5)	
SIR	6 (2.1–8.8)	6 (2.1–8.8)	6 (2.1-6)	

Data on the treatment with IT or TT at or after GKRS1 treatment, were available from 71 patients (71/81, 88%). *baseline characteristics of patients who received IT or TT without Denosumab at or after first radiosurgical treatment (GKRS1). † baseline characteristics of patients in whom the oncological therapy status was known and who did not receive IT or TT at or after GKRS1

*BM* brain metastasis, *CNS* central nervous system, *ECM* extracranial metastases, *fRT* fractionated radiotherapy, *GI* gastrointestinal, *GKRS* Gamma Knife Radiosurgery, *GPA* graded prognostic assessment, *IT* immunotherapy, *KPS* Karnofsky performance status scale, *RPA* recursive partitioning analysis, *SIR* Score Index for Radiosurgery, *TT* targeted therapy, *WBRT* whole brain radiation therapy.

### Radiosurgical technique

The radiosurgical treatments were planned with GammaPlan® (Elekta, Stockholm, Sweden) and performed with Leksell Gamma Knife® Perfexion™ (Elekta, Stockholm, Sweden). Planning sequences were performed on a 1.5 or 3.0 Tesla magnetic resonance imaging (MRI) and always included Gadolinium contrast-enhanced T1-weighted MRI sequences. Planning was performed as we have described in detail before [12, 26]. GKRS parameters are summarized in Table 2. In 56/81 (69%) patients only one GKRS treatment was performed, with 14 patients being treated by a boost dose (reduced dose). Of the remaining 25 patients, 12/81 (15%) had multiple GKRS treatments due to new BM and 13/81 (16%) received dose-staged GKRS treatments as described before [12, 26]. At GKRS1, a median of 2 BM (range: 1–10 BM) were treated in each patient. Including all GKRS treatments per patient, 264 BM were treated in 116 GKRS procedures with a median of 2 BM (range: 1–25 BM) per patient.

**Table 2:** GKRS1 parameters in patients with regular high dose, staged and boost treatment

Parameters	GKRS1 regular high dose Number of BM (*n* = 110)	Staged treatment Number of BM at first treatment (*n* = 16)	Boost treatment Number of BM (*n* = 38)
Treatment volume in cm³, median (range)	1.6 (0.1–6.1)	7.4 (2.1–13.8)	1.7 (0.2–16.6)
Isodose in %, median (range)	50 (45–90)	50 (45–50)	50 (50–60)
Prescription dose in Gy, median (range)	18.5 (17–20)	15 (10–16)	15 (10–16)
Central dose in Gy, median (range)	36 (21–44)	30 (20–36)	28 (20–32)

A total of 164 BM were treated at GKRS1. Table 2 gives an overview of radiosurgical parameters per BM at GKRS1

*BM* brain metastases, *GKRS1* first Gamma Knife radiosurgery

### Neuroradiological definitions

As standard, clinical and neuroradiological follow-up of all radiosurgically treated patients were performed every three months. We defined metastases as contrast-enhanced tumor lesions and tumor progression according to the response assessment in neuro-oncology (RANO) criteria [27]. Intralesional hemorrhage, radiation reaction and radiation necrosis were defined according to Heit et al. and Stockham et al. [28–30]. All follow-up MRIs of patients with potential postinterventional complications were reviewed by a senior neuroradiologist (W.M.), who was blinded to the clinical follow-up data.

### Statistical analysis

Statistical analysis was performed with SPSS software version 26.0 (IBM, Armonk, NY, USA).

Categorical data were presented as counts and percentages, and continuous parameters as median and range. The chi-square, Mann-Whitney U, and Wilcoxon signed-rank tests were performed as statistically appropriate. Median survival after the first GKRS was estimated by the Kaplan-Meier method and compared using Mantel-Cox log-rank test. We performed univariate followed by multivariate Cox proportional hazard regression analyses to assess if sex, KPS group (< 80% vs. ≥80%), age group (≤ 65 vs. >65 years), number of BM at GKRS1 (single vs. multiple), RPA class, ECM status at BM diagnosis, GKRS1 treatment mode (boost, staged or regular high dose) and IT or TT status at or after GKRS1 predicted survival after GKRS1. A two-sided probability value of < 0.05 was considered as statistically significant for all performed tests.

## Results

### Overall survival and outcome

The median time between the diagnosis of GI cancer and BM was 23 months (range: 0-107 months). Only one patient (1/81, 1%) was truly lost to follow-up. From the remaining 80 patients (80/81, 99%) survival data were available. The vast majority of patients (76/81; 94%) had succumbed to their disease at time of last follow-up.

The median overall survival after the diagnosis of GI cancer, the diagnosis of the initial BM and the first GKRS treatment was 35.3 months (95% confidence interval (CI) = 22-48.5), 7.5 months (95% CI = 5.8–9.2) and 6.4 months (95% CI = 4.5–8.3), respectively.

**Fig. 1:**
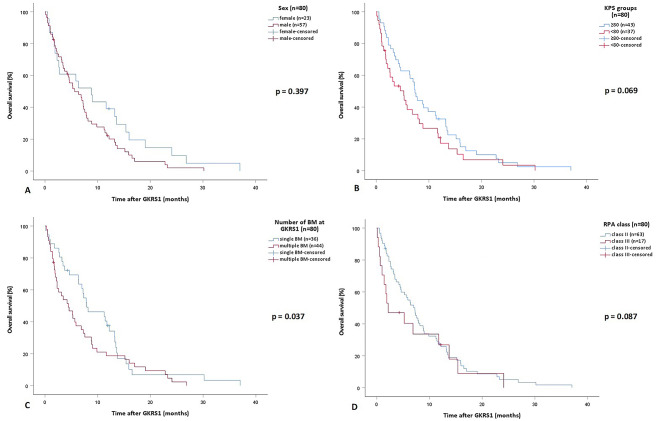
Kaplan-Meier curves showing the differences of the overall survival after GKRS1 between different treatment groups. **A** There is no difference in the median overall survival between female (8.8 months, 95% CI = 3.9-13.7) and male patients (5.7 months, 95% CI = 2.8-8.5; *p*=0.397). **B** Even though a trend towards a longer median overall survival in patients with a KPS ?80% (7.4 months, 95% CI = 6.3-8.4) compared to those with a KPS <80% (5.2 months, 95% CI = 2.2-8.3) could be observed, the results were statistically not significant (*p*=0.069). **C** Patients with a single BM (7.9 months, 95% CI = 3.3-12.5) at GKRS1 had a statistically significant longer median overall survival after GKRS1 compared to patients with multiple BM (4.4 months, 95% CI = 1.8-7.1, *p*=0.037). **D** A trend towards a longer median overall survival in patients with RPA class II (7.2 months, 95% CI = 5.4-9.1) compared to patients with RPA class III (2.2 months, 95% CI = 0-6.6) could be observed, however, the results were statistically not significant (*p*=0.087). *BM* brain metastasis, *CI* Confidence Interval, *GKRS* Gamma Knife Radiosurgery, *KPS* Karnofsky performance status scale, *RPA* Recursive partitioning analysis

After GKRS1, there were no differences in overall survival between female and male patients (*p* = 0.397, Fig. 1A). Patients with a KPS ≥ 80% showed a slightly longer median overall survival after GKRS1 compared to those with a KPS < 80% (*p* = 0.069, Fig. 1B).

We subsequently compared the median overall survival between patients ≤ 65 and > 65 years. The estimated median overall survival after GKRS1 in the ≤ 65 years group (6.4 months, 95% CI = 3.1–9.7) appeared to be similar compared to the > 65 years group (5.9 months, 95% CI = 2.8-9, *p* = 0.709).

The status of ECM at diagnosis of brain metastases did not affect the estimated median overall survival after GKRS1 (patients with ECM: 6.9 months, 95% CI = 5-8.7; patients without ECM: 3.4 months, 95% CI = 1.1–5.8, *p* = 0.820).

Patients with the largest BM ≤ 6 cm³ (6.9 months, 95% CI = 4.3–9.4) had an almost similar median overall survival compared to patients with at least one BM > 6 cm³ (5.3 months, 95% CI = 0.6–10, *p* = 0.711) at GKRS1. However, patients with a single BM at GKRS1 had a statistically significant longer median overall survival after GKRS1 compared to patients with multiple BM (*p* = 0.037, Fig. 1C).

According to the RPA classification system, 64/81 (79%) and 17/81 (21%) patients were rated as class II and III, respectively. There were no patients with RPA class I. We could not find any statistically significant differences in the median overall survival after GKRS1 between patients with RPA class II and patients with RPA class III (*p* = 0.087, Fig. 1D).

Notably, regarding the treatment dose at GKRS1, patients with regular high dose (7 months, 95% CI = 5.1–8.9), staged dose (11.4 months, 95% CI = 0-25.2) and boost dose (4.6 months, 95% CI = 4.2-5, *p* = 0.345) showed a similar median overall survival after GKRS1 compared to each other.

Finally, we compared the actual survival of our patients to the predicted survival by prognostic scores and could show that our patients had a significantly longer survival after GKRS1, compared to the general GPA (*p* < 0.0001), specific GPA (*p* < 0.001), RPA (*p* < 0.0001) and SIR (*p* < 0.008).

### Outcome and survival after GKRS1 in relation to IT or TT

Overall, 61/71 (86%) patients received chemotherapy before or at GKRS1 treatment. Data on the treatment with IT or TT at (± 30 days) and after (> 30 days) GKRS1 treatment, were available from 71 patients (71/81, 88%).

In regard to the concomitant oncological treatment at or after GKRS1, 21/71 (30%) patients received IT or TT (Table 3) and 50/71 (70%) patients did not receive any IT or TT. All patients (21/21, 100%) from the IT or TT group also received chemotherapy before or at GKRS1. Both groups showed similar baseline characteristics, except for age with significantly younger patients in the “IT or TT group” (*p* = 0.011, Table 1).

**Table 3:** Patients with IT or TT at or after GKRS1

IT or TT at or after GKRS1 “IT or TT Group” (*n* = 21)	Number of Patients (%)
Bevacizumab ¹	10 (47%)
Cetuximab ²	4 (19%)
Panitumumab ²	2 (9%)
Sunitinib ³	1 (5%)
Nivolumab^4^	1 (5%)
Trastuzumab^5^	1 (5%)
Regorafenib^6^ + Bevacizumab ¹	1 (5%)
Panitumumab ² + Bevacizumab ¹	1 (5%)

¹ *VEGF-A* vascular endothelial growth factor A inhibitor, ² *EGFR* epidermal growth factor receptor inhibitor, ³ RTK receptor tyrosine kinase inhibitor, ^4^*PD-1* programmed cell death-1 inhibitor, ^5^*HER2* human epidermal growth factor receptor 2, ^6^*RTK* receptor tyrosine kinase inhibitor

*GKRS1* first Gamma Knife radiosurgery, *IT* immunotherapy, *TT* targeted therapy

**Fig. 2:**
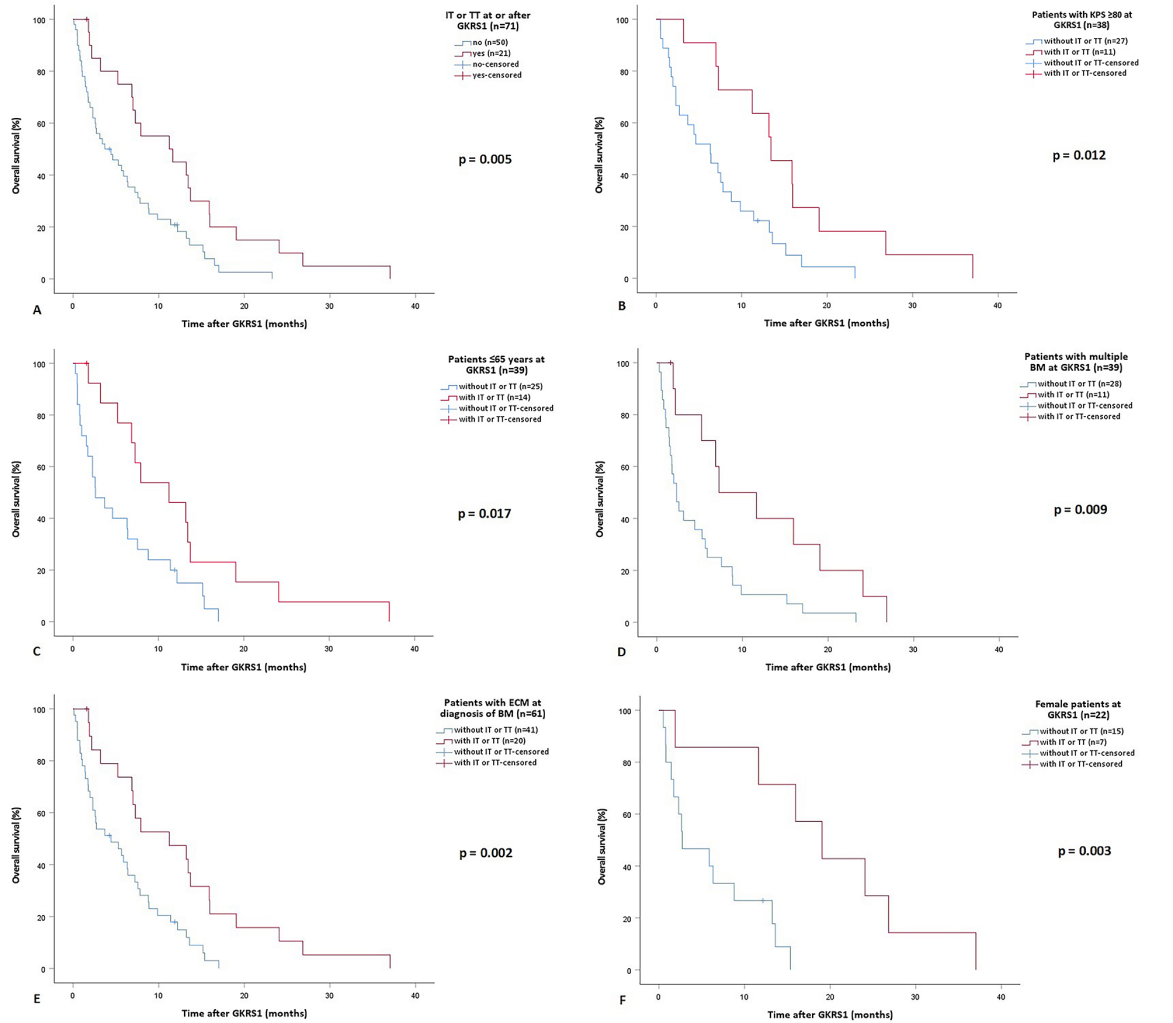
Kaplan-Meier curves showing the overall survival after GKRS1 between different groups in relation to IT or TT. **A** Patients with IT or TT at or after GKRS1 had a significantly longer survival after GKRS1 (21/70 or 30%, 11.2 months, 95% CI = 3.1–19.4) compared to patients without IT or TT at or after GKRS1 (50/71 or 70%, 3.7 months, 95% CI = 0.8–6.6, *p* = 0.005). **B** Among patients with KPS ≥ 80%, patients with IT or TT (13.4 months, 95% CI = 8.4–18.5) showed a significantly longer median overall survival after GKRS1 compared to those without IT or TT (6.3 months, 95% CI = 3-9.7, *p* = 0.012). **C** In the group of patients ≤ 65 years, those with IT or TT at or after GKRS1 (11.2 months, 95% CI = 4.3–18.2) showed a significantly longer median overall survival compared to patients without IT or TT (2.6 months, 95% CI = 0.3–4.9, *p* = 0.017). **D** Analyzing patients with multiple BM at GKRS1, we could show that those with IT or TT at or after GKRS1 (7.3 months, 95% CI = 0-14.6) showed a significantly longer median overall survival after GKRS1 compared to patients without IT or TT (2.3 months, 95% CI = 1.2–3.4, *p* = 0.009). **E** Among patients with ECM at diagnosis of BM, those with IT or TT at or after GKRS1 (11.2 months, 95% CI = 2.8–19.7) showed a significantly longer median overall survival after GKRS1 compared to patients without IT or TT (4.4 months, 95% CI = 0.7–8.1, *p* = 0.002). **F** Among female patients, those with IT or TT at or after GKRS1 (19.1 months, 95% CI = 11.1–27) showed a significantly longer median overall survival after GKRS1 compared to patients without IT or TT (2.7 months, 95% CI = 0-7.2, *p* = 0.003). *BM* brain metastasis, *CI* Confidence Interval, *ECM* extracranial metastases, *GKRS* Gamma Knife Radiosurgery, *IT* immunotherapy, *KPS* Karnofsky performance status scale, *TT* targeted therapy

We could show that patients with IT or TT at or after GKRS1 had a significantly longer survival after GKRS1 compared to patients without IT or TT at or after GKRS1 (*p* = 0.005, Fig. 2A).

Even after excluding patients with a KPS of < 80%, we could show that the survival after GKRS1 remained significantly longer in patients with IT or TT compared to patients without IT or TT (*p* = 0.012, Fig. 2B).

In addition, a sub-analysis only among younger patients ≤ 65 years revealed that, patients treated with IT or TT had a statistically significant longer estimated median overall survival after GKRS1 compared to patients without IT or TT (*p* = 0.017, Fig. 2C).

In the older patient group > 65 years, those with IT or TT (11.6 months, 95% CI = 0-23.5) did not show any statistically significant differences in the estimated median overall survival after GKRS1 compared to patients without IT or TT (5.3 months, 95% CI = 1.9–8.7, *p* = 0.158).

Even among patients with multiple BM at GKRS1, the estimated median overall survival after GKRS1 remained significantly longer in patients with IT or TT compared to those without IT or TT (*p* = 0.009, Fig. 2D).

Same results applied to the positive ECM status at diagnosis of BM, as patients with IT or TT had a longer median overall survival after GKRS1 compared to those without IT or TT (*p* = 0.002, Fig. 2E).

By analyzing the median overall survival separately in the female and male group, we could also show that female patients with IT or TT showed a statistically significant longer estimated median overall survival after GKRS1 compared to female patients without IT or TT (*p* = 0.003, Fig. 2F). In the male group, patients with IT or TT (7.3 months, 95% CI = 6-8.5) did not show any statistically significant differences in estimated median overall survival after GKRS1 compared to patients without IT or TT (4.4 months, 95% CI = 2-6.9, *p* = 0.091).

Consequently, we calculated univariate Cox regression analyses evaluating sex, KPS group, age group, number of BM at GKRS1, RPA class, ECM status at BM diagnosis, GKRS1 treatment mode (boost, staged or regular high dose) and IT or TT status at or after GKRS1 as survival predictors among the total sample. Univariate Cox regressions showed that IT or TT at or after GKRS1 had a significant influence on the OS (HR: 2.227, 95% CI 1.261–3.935, *p* = 0.006).

Next, for the 71 patients of whom data on IT or TT were available, we included all covariates into a multivariate Cox regression model (sex, KPS group, age group, number of BM at GKRS1, RPA class, ECM status at BM diagnosis, GKRS1 treatment mode (boost, staged or regular high dose) and IT or TT status at or after GKRS1). Consequently, only IT or TT at or after GKRS1 was revealed as an independent predictor for a longer survival after GKRS1 (HR: 2.221, 95% CI 1.257–3.925, *p* = 0.006).

### Tumor control and Complications after GKRS in relation to IT or TT

For the assessment of post-radiosurgical complications, clinical and radiological follow-up data from 58 patients (58/81, 72%) were available. One patient from the IT or TT group and 15 patients without IT or TT died before the first clinical follow-up. After excluding all patients without an available neuroradiological follow-up, we evaluated the follow-up MR images of 58 patients.

The Kaplan-Meier estimated mean time until local progression was 20 months (95% CI = 17.4–22.6). There was no significant difference of the Kaplan-Meier estimated mean time until local progression between patients who received IT or TT (18.7 months, 95% CI = 14.6–22.9) and patients without IT or TT (21.3 months, 95% CI = 18.7–23.9, *p* = 0.637).

There were no statistically significant differences in regard to the progression rate (*p* = 0.169), hemorrhage rate (*p* = 0.352) and radiation reaction or necrosis (*p* = 1.000) between patients with IT or TT and those without IT or TT.

Radiation reaction and necrosis after GKRS1 were observed in 6/58 (10%) and 1/58 (2%) patient, respectively. Intralesional hemorrhage was diagnosed in one patient (2%). Progression after GKRS1 was observed in 7/58 (12%). One patient (2%) with progression was subsequently treated by whole brain radiation therapy (WBRT) and further 3 patients (5%) underwent microsurgical resection. The remaining 3 patients had no further BM treatment.

## Discussion

### Prognostic factors for survival after radiosurgery among GI BM patients

In recent years, the treatment of BM patients with IT or TT and concomitant radiosurgery has improved the survival after radiosurgery in patients with BM from non-small cell lung cancer or melanoma [12–16]. Some studies on non-small cell lung cancer or melanoma even suggest that the radiosurgical treatment with concurrent IT has a positive synergetic effect on the systemic progression of primary malignancies via the abscopal effect [31]. Still, most of GI cancer patients are in a palliative care setting at the time of BM diagnosis [7]. In this study, we report the outcome of radiosurgically treated BM cancer patients from GI and associated primary cancers. As others have done before, we included BM patients with pancreatic and liver cancer as well, since the response to radiosurgical treatment is comparable among BM from these primary tumors [3, 19–22, 32].

In a first step, we could show that the actual overall survival after GKRS was significantly longer among all our patients compared to the predicted overall survival according to calculated prognostic scores [6, 23–25]. Nonetheless, previously published scores do not consider contemporary systemic oncological treatment options [6, 23–25, 33]. Subsequently, we aimed to analyze which factors affect the overall survival after radiosurgery in our patient cohort.

We could observe a trend towards a longer median overall survival after GKRS1 in patients with a KPS ≥ 80% and with RPA class II. These results were statistically not significant, which is in contrast to some former studies and might be caused by our sample size [5, 7, 19, 32, 34, 35]. It could also indicate that factors such as KPS, age, ECM status at GKRS1 do not represent the most important predictors on the overall survival after GKRS.

In contrast, a significantly longer overall survival after GKRS1 was observed in patients treated with concomitant IT or TT compared to patients without IT or TT. Of note, the vast majority of these patients received TT treatment and only a limited number of patients in our study was treated with IT. However, this clear benefit in survival after GKRS of patients treated with concomitant GKRS and mainly targeted therapy remained evident even in the sub-group analyses of patients with a KPS ≥ 80%, of patients aged ≤ 65 years and among female patients alone. Moreover, IT or mainly TT treatment at or after GKRS1 was revealed as the only independent predictor for a longer survival after GKRS1 among GI BM patients in a multivariate analysis that included sex, KPS score, age, number of BM, RPA class, ECM status, GKRS treatment mode and IT or TT treatment status. Our results are in accordance with previously published studies, which demonstrated a clear benefit of IT or TT on the survival after radiosurgery among GI and other primary cancers [12, 36, 37].

Previous studies have hypothesized that sex hormones may also have an effect on tumor aggressiveness, with the male sex being associated with a poorer survival [34, 38, 39]. Thus, the impact of the concomitant radiosurgical and IT or TT treatment on the outcome in male patients should be investigated in a larger sample.

### Radiosurgery in the modern management of BM from GI cancers

Radiosurgery has established itself as the primary local treatment option for BM patients, generally achieving excellent tumor control and low complication rates [10, 11]. Compared to BM from lung or breast cancer or melanoma, BM from GI cancers are rare [4–7]. Consequently, data on the radiosurgical treatment of BM and the effect of IT or TT on the outcome after GKRS in GI cancer patients are scarce [17, 18, 37, 40]. However, the incidence rates of GI cancer and subsequently BM from GI cancer are rising [1, 3, 5–7, 41].

The treatment of BM from GI cancers remains a challenge since by the time BM are diagnosed, the overall prognosis is generally poor, they are often difficult to control and regularly react with persistent peritumoral edema after radiosurgical treatment [19, 21]. Consequently, the modern management of patients with BM from GI cancers requires a multimodal approach [41].

According to the treatment strategy at our department, patients with single or oligometastases with the largest tumor volume ≤ 6 cm³ received a regular high dose treatment [21]. In cases of multiple metastases (> 5 BM) a slightly reduced boost dose was mainly administered. Previous studies showed that the presence of multiple BM is a significant predictor among GI cancer patients [33, 37]. However, it is not per se a prognostic factor in modern BM management among other primary tumors [7, 42]. In our present study, the number of BM was not an independent predictor of survival after GKRS in the multivariate analysis.

Moreover, two-fraction dose-staged treatment was performed to enable the treatment of large or high-risk BM, as published before [26]. Of note, our analyses showed that there were no statistically significant differences in the outcome of patients treated with different radiosurgical strategies. Thus, we conclude that with the advancement of radiosurgical technique and oncological treatment options, the number of BM will become of less importance also among GI cancer patients and that even patients with more than 5 BM or large high-risk BM benefit from radiosurgical treatment [12, 26].

Of note, the vast majority of patients after GKRS were diagnosed with stable / regressive BM. Thus, good local tumor control rates and acceptable low rates of radiation reaction / necrosis demonstrate the safety and efficacy of GKRS in patients with BM from GI cancers under concomitant IT or TT.

However, there was no difference in BM progression between patients with and without IT or TT. This observation is supported by other data demonstrating that tumor histology, whole brain irradiation, targeted therapies, and antineoplastic therapies did not improve local tumor control in GI BM patients [19].

In summary, the combined radiosurgical and targeted oncological treatment has the potential to prolong the overall survival of BM patients from GI cancers. However, since systemic therapies, even IT or TT, are limited in their access through the blood-brain barrier and thus, may not sufficiently prevent BM progression, local treatment options, such as radiosurgery, remain of utmost importance in modern BM management [32, 33, 37, 41, 43, 44].

### Limitations

We are aware that this study has some limitations which need to be addressed. First, this study was limited by its retrospective character, with data presented only by a single institute with a limited number of patients including different types of GI and associated primary cancers. Thus, the information on the type of systemic oncological treatment was missing in some cases. Moreover, in the group of IT or TT treatment, the vast majority of patients received TT treatment and only a limited number of patients was treated with IT.

Second, although there is no statistically significant difference in most of the baseline characteristics between patients with and without IT or TT, patients with IT or TT were significantly younger than patients without IT or TT. Therefore, age could have been a major factor in choosing candidates for systemic treatment. However, our multivariate analysis including age revealed IT and TT as an independent predictor for a longer survival after GKRS1.

## Conclusions

Good local tumor control rates and low rates of side effects demonstrate the safety and efficacy of GKRS in patients with BM from GI cancers. The concomitant radiosurgical and targeted oncological treatment significantly improves the survival after GKRS without increasing the rate of adverse radiation effects. To provide local tumor control, radiosurgery remains of utmost importance in modern GI BM management.

## Data Availability

Research data will not be shared.

## Abbreviations

BM: Brain Metastases
CI: Confidence Interval
ECM: Extracranial Metastases
GI: Gastrointestinal
GKRS: Gamma Knife Radiosurgery
GKRS1: First Gamma Knife Radiosurgery
GPA: Graded Prognostic Assessment
HR: Hazard Ratio
IT: Immunotherapy
KPS: Karnofsky Performance Status Scale
MRI: Magnetic Resonance Imaging
NSCLC: Non-Small Cell Lung Cancer
RPA: Recursive Partitioning Analysis
RANO: Response Assessment in Neuro-Oncology
SIR: Score Index for Radiosurgery
SRS: Stereotactic Radiosurgery
TT: Targeted Therapy
WBRT: Whole Brain Radiation Therapy
